# Tardive and spontaneous dyskinesia incidence in the general population

**DOI:** 10.1186/1471-244X-13-152

**Published:** 2013-05-28

**Authors:** Ray M Merrill, Joseph L Lyon, Paul M Matiaco

**Affiliations:** 1 Department of Health Science, Brigham Young University, 229-A Richards Building, Provo 84602, UT, USA; 2 Department of Family and Preventive Medicine, University of Utah, Salt Lake City, UT, USA

**Keywords:** Antipsychotic drugs, Dyskinesia, Incidence rates, Metoclopramide, Risk factors

## Abstract

**Background:**

To identify the incidence rate of spontaneous dyskinesia (SD) and tardive dyskinesia (TD) in a general population and to examine the association between dykinesia and potential risk factors (exposure to metoclopramide [MCP], antipsychotic drugs, and history of diabetes and psychoses).

**Methods:**

A retrospective cohort study was conducted for the years 2001 through 2010, based on medical claims data from the Deseret Mutual Benefit Administrators (DMBA).

**Results:**

Thirty-four cases of TD and 229 cases of SD were identified. The incidence rate of TD among persons previously prescribed an antipsychotic or metoclopramide (MCP) (per 1,000) was 4.6 (1.6-7.7) for those with antipsychotic drug use only, 8.5 (4.8-12.2) for those with MCP use only, and 15.0 (2.0-28.1) for those with both antipsychotic and MCP use. In the general population, the incidence rate (per 100,000 person-years) of TD was 4.3 and of probable SD was 28.7. The incidence rates of TD and SD increased with age and were greater for females. Those with diabetes or psychoses had almost a 3-fold greater risk of TD than those without either of these diseases. Persons with schizophrenia had 31.2 times increased risk of TD than those without the disease. Positive associations also existed between the selected diseases and the incidence rate of probable SD, with persons with schizophrenia having 4.4 times greater risk of SD than those without the disease.

**Conclusions:**

SD and TD are rare in this general population. Diabetes, psychoses, and especially schizophrenia are positively associated with SD and TD. A higher proportion of those with SD present with spasm of the eyelid muscles (blepharospasm) compared more with the TD cases who present more with orofacial muscular problems.

## Background

The introduction of chlorpromazine and other antipsychotic drugs into medical practice in the 1950’s revolutionized the treatment of schizophrenia. However, these drugs were not without some unanticipated side effects. The most common were increased occurrence of neuromuscular problems, especially Parkinson’s like muscle rigidity. A less common side effect was dyskinesia. Dyskinesia manifests its presence as abnormal involuntary, repetitive, persistent, stereotypic movements usually of the facial muscles but can also involve other muscle groups including the extremities and the torso [1]. When this condition occurs sometime after the initiation of antipsychotic drugs that damage GABAergic medium spiny neurons (MSNs), it is referred to as tardive (or delayed) dyskinesia (TD) [2]. New research has distinguished MSNs as the first station in the corticostriato-thalamo-cortical circuit that regulates the amplitude and velocity of movements [2].

Studies have identified an elevated risk of dyskinesia among schizophrenics exposed to first- and second-generation neuroleptic (antipsychotic) drugs, with second-generation neuroleptics posing a lower risk [3-7]. First-generation antipsychotic drugs are more likely to cause movement disorders because they bind tightly to dopaminergic neuroreceptors (e.g., haloperidol). Nevertheless, some of these earlier developed drugs are still in use today. There is currently little information as to the incidence rate of dyskinesia among those without an underlying psychosis. Other drugs, especially metoclopramide used for the treatment of gastric paresis, were also claimed to increase the incidence of TD. The common pathway for these side effects relates to the dopamine-2 receptors in the brain.

It is now recognized that antipsychotic-naïve individuals can develop involuntary movement disorders and these are classified as spontaneous dyskinesia (SD) [8-10]. Drugs associated with TD are chlorpromazine and other antipsychotic drugs and metoclopramide (MCP) [3,11,12]. Spontaneous and tardive dyskinesia are more common in older ages [13,14]. The incidence of spontaneous dyskinesia by age and sex has not been well studied. For drug-related dyskinesia, the incidence may be related to the increased use of neuroleptic drugs or MCP in older individuals. For example, MCP is used to treat gastrointestinal problems associated with diabetes mellitis. In addition, there is a strong correlation between age and cumulative antipsychotic exposure.

In addition to MCP and antipsychotic drugs, diabetes mellitus has been reported to be a risk factor for TD [15,16], although there remains some uncertainty about the causal mechanisms of this link [17]. Diabetes may be associated with TD because MCP has been used to treat gastro-paresis, a consequence of severe diabetes [18,19]. Other studies have suggested an independent association between dyskinesia and diseases such as diabetes, schizophrenia, and other psychoses in the absence of ingestion of MCP or antipsychotic [8-10]. While the focus in this paper is on first-generation antipsychotics and dyskinesia, newer second-generation antipsychotics (e.g., clozapine and olanzapine) have been shown to cause problems related to metabolic syndrome, thereby contributing to obesity and type 2 diabetes mellitus [20].

Prevalence and incidence rates of TD and SD have varied widely because of differences in patient characteristics and methodological approaches. Most studies have used cross-sectional (or prevalence) data on patients with schizophrenia or other psychoses, but some have used retrospective cohort data and a small number have used prospective cohort data [3,4,8-10,21,22]. The purpose of the current study was to determine the incidence of all types of dyskinesia in general population and also to examine its connection to underlying medical conditions such as diabetes independent of antipsychotic medication use, and in association with use of first-generation antipsychotic drugs and metoclopramide.

## Methods

### Study population

A retrospective cohort study was conducted using data from the Deseret Mutual Benefit Administrators (DMBA), a health insurance company for employees of the Church of Jesus Christ of Latter-day Saints. The company was established in 1970 to provide health insurance and retirement income to church employees and their families. There are about 80,000 individuals covered by the DMBA each year. There is little employee turnover, estimated at less than 5% per year [23]. The majority of turnover occurred among young adults who lose eligibility for coverage under their parents’ health insurance plan and individuals who become eligible for Medicare. Membership in the church is a requirement for employment, with two exceptions; employees of Deseret management companies and commercial enterprises do not need to be members of the church (roughly 11-13% of enrollees). The majority (70%) of enrollees work in Utah. Approximately 45.6% of enrollees are employed in the Latter-day Saint Church education system principally as teachers; 21.1% of enrollees are maintenance and custodial workers; and the remaining 33.3% work in other capacities for the Church. There is a relatively low prevalence of tobacco smoking, alcohol consumption, and illicit drug use among members of the database due to the proscription of these substances by the Latter-day Saint Church [24].

Employees, spouses and dependent children are included in the database. During the study period 2001 through 2010, there were 144,308 distinct individuals in the database and they accrued 798,542 person-years of observation. Mean age increased from 34.7 (SD = 23.3) in 2001 to 37.1 (SD = 24.6) in 2010, with 49.9% ages 0–24, 20.6% ages 25–44, 19.0% ages 45–64, and 10.5% ages 65 years and older. The percentage of males was slightly lower than females (49.2 vs. 50.8%). At age 65, many move to Medicare, but purchase supplemental insurance through DMBA. The supplemental insurance should capture all new claims for these individuals.

The database was de-identified according to Health Insurance Portability and Accountability Act (HIPAA) guidelines and was exempt from the need for informed consent by the Institutional Review Board at the University of Utah. The current study was approved and classified as a low risk study by the Institutional Review Board.

### Outcome variables

The primary outcome variables of interest were tardive dyskinesia (TD) and spontaneous dyskinesia or dystonia (SD).

Tardive dyskinesia and SD were identified by any one (or a combination) of the International Classification of Diseases, 9th Revision (ICD-9), codes 333.81, 333.82, 333.83, 333.84, 333.85 (a new code introduced October 1, 2006), and 333.89 being present on a paid DMBA claim as a reason for the enrollee’s office visit [25]. If a claim was filed for use of any antipsychotic drug and/or MCP prior to the claim for dyskinesia, the disease was classified as TD, otherwise as SD. The ICD coding system did not include a separate code for a dyskinesia that appeared after exposure to medication until October 2006 when the code 333.85 was then introduced. For this study we reviewed each individual’s prescription drug use and only included those with prior history of antipsychotic or MCP use before the diagnosis of any type of dyskinesia. We then combined those with prior drug exposure with any of the dyskinesia codes with the new 333.85 code. Among 263 patients with one of the ICD-9 codes for a specific dyskinesia, 34 were classified as TD and 229 as SD.

In this study, we defined a person as having “probable TD” or “probable SD” if he/she had one or more claims involving the above ICD-9 codes (and met the medication use criteria above). We also present results with a stricter definition of TD and SD, wherein at least two claims were paid for the dyskinesia within a 12 month time period. Our reasoning was that a physician may make a tentative diagnosis based on a single visit by a patient with a complaint of unexplained involuntary muscular movements. If symptoms persisted, then this would cause the individual to make a second visit, and so two visits within one year with the same diagnosis would be more likely to identify a patient with actual disease. This definition better fits the Schooler-Kane criteria for the diagnosis of TD or SD that only accepts a diagnosis based on two separate examinations three months apart [26]. The confidentiality agreement that covers our use of the DMBA database does not allow us to characterize physicians by their medical specialty so it was not possible to identify how many diagnoses were made by a primary care physician and how many were made by a neurologist, or psychiatrist.

### Antipsychotic

An antipsychotic is any drug used to treat psychosis, particularly schizophrenia. For the purpose of this study, we restricted our analysis to the classic antipsychotic drugs, which were introduced into psychiatric practice in the 1950’s and were still in use between 2001 and 2010. Classical antipsychotic drugs have a proven or suspected association with TD. Antipsychotic drugs were chosen from lists of classic drugs used during 2001 through 2010 [27,28]: amitriptyline, chlorpromazine, compazine, etrafon, fluphenazine, haldol, haloperidol, halperon, kenazine, loxapine, loxitane, maxolon, melacen, mellaril, mesoridazine besylate, millazine, moban, navane, orap, ormazine, perphenazine, prochlorperazine, serentil, stalizin, thioridazine, triavil, trifluoperazine, trilafon, trifluoperazine. Use of these drugs was identified using corresponding National Drug Codes (NDCs), and ranged in dose from 1 mg to 200 mg. In a study involving psychiatric patients over 45 years of age, use of antipsychotic drugs greater than 30 days was considered to be associated with higher risk for TD [29]. Antipsychotic drug use had to precede TD in order to be considered a cause of the disease.

### Metoclopramide

Researchers have recommended that MCP should not be used for more than 3 months in order to avoid the risk of TD [26]. More recently the Food and Drug Administration (FDA) has recommended that MCP be used for no longer than 30 days to avoid the risk of TD [30]. Both oral and non-oral MCP products are available in the US, each with a unique drug code. The non-oral MCP products include injections and topical preparations. Drug lists were queried to find the oral and injection products of MCP. The National Drug Code (NDC), and identification number issued by the FDA, is the reference used in the DMBA database. The following national drug current MCP products, as well as products withdrawn during the analysis period, were used in the study: metoclopramide, metoclopramine, metozolv, myclopramide, octamide, raglan. These medications ranged in dose from 5 mg to 10 mg. Topical MCP was not used in assessing MCP exposure because transdermal absorption is low [31,32].

### Diabetes diagnosis

Diabetics are at increased risk for gastroparesis and MCP has been the preferred drug to treat this condition [19]. Both type I and II diabetes mellitus were included in the study. The ICD-9 code 250 was used to identify a case. Hyperglycemia NOS (ICD-9 codes 790.6), neonatal diabetes mellitus (775.1), nonclinical diabetes (790.2), and diabetes complicating pregnancy, childbirth, or the puerperium (648.0) were excluded. Diabetes was only associated with dyskinesia if it was diagnosed prior to TD or SD.

### Psychoses

Individuals with psychoses may be at increased risk of spontaneous movement disorders, even without a history of MCP or antipsychotic drug use [6,7,33,34]. The ICD-9 code 295 was used to identify persons with schizophrenia, and codes 296–299 were used to identify other psychoses: affective psychoses (ICD-9 = 296), paranoid states (ICD-9 = 297), other nonorganic psychoses (ICD-9 = 298), and psychoses with origin specific to childhood (ICD-9 = 299). Schizophrenia and other psychoses were only associated with dyskinesia if they were diagnosed prior to TD or SD.

### Statistical techniques

Counts, percentages, and incidence rates were used to describe the data. The incidence rates reflected new cases in the numerator. A table was constructed to calculate the time under observation of each of the individuals included in this study since there were loses and additions of individuals during the 10 years of the study. Each person’s contribution of observable time was calculated based on year end enrollment data and an overall person-time denominator was obtained. We also calculate person-time for those with diabetes, schizophrenia or other psychoses. This allowed us to estimate incidence rates for each of these conditions. Rate ratios were used to measure the association between dyskinesia and the selected diseases and types of medications used. Ninety-five percent confidence intervals were calculated for the rates and rate ratios. Rate ratios were considered significant if the confidence interval did not overlap 1. Statistical significance was based on the two-sided hypothesis and a P value of 0.05 or less. Analyses were performed using SAS 9.3 (SAS Institute Inc., Carry, NC, 2002–2010).

## Results

**Figure 1:**
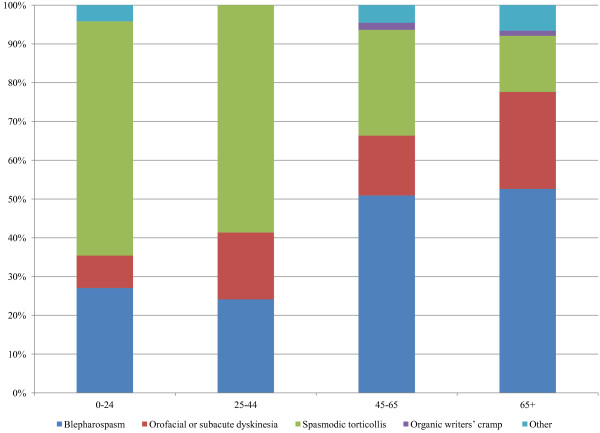
**Distribution of ICD-9 classifications of dyskinesia according to age group.** Source: Deseret Mutual Benefit Administrators, 2001–2010. Note: Based on TD and SD combined. The distribution significantly differed by age group, P < 0.0001.

During 2001 through 2010, 263 individuals in this population had a first time diagnosis of dyskinesia. The distribution of ICD-9 codes used to define TD and SD are presented in Table 1. The distribution of codes differed between TD and SD, with those who developed TD presenting with a greater percentage of orofacial or subacute dyskinesia and those with SD experiencing more blepharospasm. For TD and SD combined, the distribution of codes did not significantly differ between men and women, but varied by age (Figure 1).

**Table 1:** Counts of dyskinesia according to ICD-9 classification

**ICD-9 codes**	**Description**	**No.**	**%**	**No.**^**a**^	**%**^**a**^
	Tardive dyskinesia – drug related …				
333.81	Blepharospasm	11	32.4	8	32.0
333.82/333.85	Orofacial or subacute dyskinesia	11	32.4	8	32.0
333.83	Spasmodic torticollis	10	29.4	7	28.0
333.89	Other	2	5.8	2	8.0
		34		25	
	Spontaneous dyskinesia – non-drug related …				
333.81	Blepharospasm	105	45.8	67	45.6
333.82	Orofacial or subacute dyskinesia	34	14.8	26	17.7
333.83	Spasmodic torticollis	77	33.6	48	32.6
333.84	Organic writers’ cramp	3	1.3	2	1.4
333.89	Other	10	4.4	4	2.7
		229		147	

Source: Deseret Mutual Benefit Administrators, 2001–2010.

^a^Two or more diagnoses within a 12 month time period.

The number of enrollees prescribed antipsychotic drugs only, MCP only, or both are shown in Table 2. The table also shows the number with an ICD-9 claim (333.81-333.89) following prescribed medication. The right portion of the table is restricted to those cases with two or more diagnoses within a 12 month time period. The rate of TD is lowest for those treated with antipsychotic drugs only and greatest for those treated with both types of medications prior to their diagnoses. Prior antipsychotic and/or MCP use tended to be at least three months prior to diagnosis. Results are similar under the case requirement of two or more diagnoses within a 12 month time period.

**Table 2:** Tardive Dyskinesia (TD) by prior use of Antipsychotic and/or Metoclopramide

**Medication**	**No. of enrollees prescribed medications**	**No. with ICD-9 Claim 333.81-333.89 within 3 months of medication use**	**No. with ICD-9 Claim 333.81-333.89 not within 3 months of medication use**	**Rate of TD per 1,000 treated with medication**	**95% CI**	**No. with ICD-9 Claim 333.81-333.89 within 3 months of medication use**^**a**^	**No. with ICD-9 Claim 333.81-333.89 not within 3 months of medication use **^**a**^	**Rate of TD per 1,000 treated with medication **^**a**^	**95% CI**^**a**^
Antipsychotic only	1,942	0	9	4.6	1.6-7.7	0	5	2.6	0.3-4.8
Metoclopramide only	2,352	3	17	8.5	4.8-12.2	3	13	6.8	3.5-10.1
Both Medications	333	3	2	15.0	2.0-28.1	2	2	12.0	0.3-23.7

Source: Deseret Mutual Benefit Administrators, 2001–2010.

^a^Two or more diagnoses within a 12 month time period.

**Figure 2:**
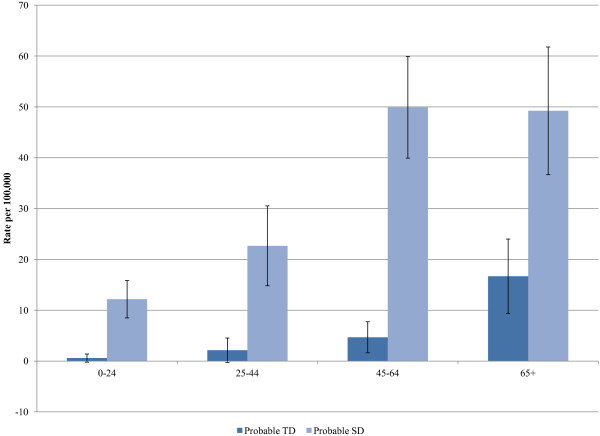
**Incidence of Tardive Dyskinesia (TD) and Spontaneous Dyskinesia (SD) according to age.** Source: Deseret Mutual Benefit Administrators, 2001–2010.

**Figure 3:**
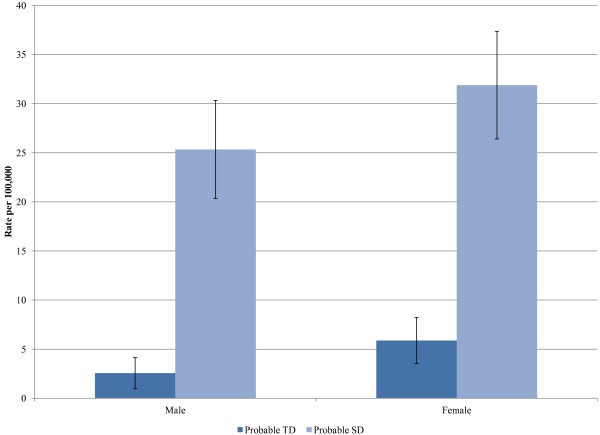
**Incidence of Tardive Dyskinesia (TD) and Spontaneous Dyskinesia (SD) according to sex.** Source: Deseret Mutual Benefit Administrators, 2001–2010.

Incidence rates of probable TD and SD, along with incidence rates based on the more restricted definition are shown in Table 3. The rates of SD are about 6 to 7 times greater than for TD. The incidence rates of TD and SD increased with age (Figure 2) and were greater for females (Figure 3). There were no statistically significant changes in the incidence rates of TD or SD across calendar years.

**Table 3:** Incidence rates of Tardive Dyskinesia and Spontaneous Dykinesia

	**Rate per 100,000 person-years**	**95% CI**	**Rate per 100,000 person-years**^**a**^	**95% CI **^**a**^
Tardive dyskinesia	4.3	2.8-5.7	3.1	1.9-4.4
Spontaneous dyskinesia	28.7	25.0-32.4	18.4	15.4-21.4

Source: Deseret Mutual Benefit Administrators, 2001–2010.

^a^Two or more diagnoses within a 12 month time period.

Positive associations existed between selected diseases (diabetes, schizophrenia, and other psychoses) and the incidence rate of probable TD (Table 4). Those with diabetes or psychoses had almost a 3-fold greater risk of TD than those without either of these diseases. Persons with schizophrenia had 31.2 times increased risk of TD than those without the disease (4.4 times the risk for those with no prior use of an antipsychotic and/or metoclopramide). Positive associations also existed between the selected diseases and the incidence rate of probable SD. Diabetes had a slightly higher association with the incidence rate of SD than with TD, and psychoses had a smaller association with the incidence rate of SD than with TD, albeit statistically significant. Persons with schizophrenia had 4.4 times greater risk of SD than those without the disease, a much smaller rate ratio than in the case of TD.

**Table 4:** Rate ratio of Tardive Dyskinesia (TD) Spontaneous Dyskinesia (SD) according to diabetes, Schizophrenia, and other Psychoses

		**Probable TD**^**a**^			**Probable SD**^**b**^		
	**Person years**	**No.**	**Rate ratio**	**95% CI**	**No.**	**Rate ratio**	**95% CI**
Diabetes							
Yes	66758	7	2.8	1.2-6.5	37	3.0	2.1-4.3
No	731784	27			135		
Schizophrenia							
Yes	1597	2	31.2	7.5-130.1	2	4.4	1.1-17.7
No	796945	32			227		
Psychoses^c^							
Yes	59252	6	2.7	1.1-6.5	26	1.6	1.1-2.4
No	739290	28			203		

Source: Deseret Mutual Benefit Administrators, 2001–2010.

Diabetes (ICD-9-CM codes 250- < 251) only counted if it occurred prior to TD.

Psychosis (ICD-9-CM codes 296- < 300) only counted if it occurred prior to TD.

Schizophrenia (ICD-9-CM codes 295- < 296) only counted if it occurred prior to TD.

^a^Claim filed with an ICD-9 code 333.81-333.89, with prior use of an antipsychotic and/or metoclopramide.

^b^Claim filed with an ICD-9 code 333.81-333.89, with no prior use of an antipsychotic and/or metoclopramide.

^c^5 of the 6 counts for probable TD and 24 or the 26 for probable SD involved affective psychoses (ICD-9 296).

## Discussion

We believe this is the first report of the incidence of spontaneous and tardive dyskinesia in a general population. The strengths of the study include a stable population, a ten year period of observation, an accurate measurement of the population at risk, the ability to identify prior diseases from the same database, the ability to link to pharmacy records, stable and experienced physician providers, and a low percentage of tobacco smokers.

Others have made estimates of the incidence of TD. Wiholm and colleagues identified 11 new cases of TD (1 in 2000–2800 treatment-years) in the Swedish population over a five year time period and linked these to use of MCP using reports from an adverse drug reaction reporting system that relied on self-reports by physicians [9]. Their study did not provide information about the incidence of SD, but the authors suggested there was an association between long term use of MCP and the development of TD, because many of those with TD had long term use of MCP. Bateman and colleagues reported the incidence of dystonia and dyskinesia to be 28.6 per million prescriptions of MCP in the United Kingdom, also based on an adverse drug reaction reporting system [35]. The incidence of dystonia and dyskinesia was 1.8 times greater in females than males.

Glazer and colleagues estimated the incidence of TD among a group of patients with schizophrenia confined to a large residential psychiatric hospital after five years of antipsychotic exposure (32%), 15 years of exposure (57%), and 25 years of exposure (68%) [36]. The study did not report information about dyskinesia among those without schizophrenia, nor was information available about dyskinesia among those not exposed to antipsychotic drugs. Inada and colleagues calculated an annual incidence rate of TD among psychiatric patients from 11 facilities in Japan [14]. The study found an annual incidence rate of TD to be 3.7%. Dyskinesia was not reported according to antipsychotic status. None of these studies were controlled for tobacco smoking, which has been suggested to be an independent cause of dyskinesia [37,38].

These studies are not directly comparable with our study because they tended to focus on individuals continuously exposed to MCP or high doses of antipsychotic drugs among individuals with schizophrenia. Very few in our study likely had long-term exposure to these medications. In addition, their populations involved psychiatric patients severe enough to require long-term institutionalization, whereas none of those with schizophrenia in our population had disease severe enough to be institutionalized. Finally, our population likely had far fewer smokers because tobacco smoking and alcohol drinking are proscribed by the Latter-day Saint Church [24].

Most other studies have measured the prevalence, not the incidence, of TD. The initial studies measured the prevalence of TD among those committed to psychiatric hospitals for schizophrenia or among patients in extended care facilities with various psychoses [4]. A study by Ganzini and colleagues in 1993 was the first to estimate the prevalence of TD among non-psychiatric outpatients treated for three months or longer with MCP and who received their medical care through a Veterans hospital [10]. The high prevalence of TD among those exposed to MCP compared with the control group (29% versus 16%) led the authors to suggest that TD and SD were common conditions in the general population, and that both drug-induced and spontaneous dystonias were much more serious medical problem than had previously been recognized. A study by Sewell and colleagues, who based their study of TD on veterans receiving medical care at the San Diego VA Medical Center, came to a similar conclusion [39].

Our study findings differ from those reported in these two earlier papers. Specifically, the incidence rates presented in the current study do not support prevalence rates of 12% to 29% for SD or TD in the general population. A crude estimation of our period prevalence rates based on about 80,000 individuals under observation during each of the 10 years of the study, and cumulating all our cases with two or more visits yields a 10 year period prevalence of 1.8 per 1,000 individuals. For all those using the antipsychotic drugs and MCP, we have a prevalence of 6.1 per 1000. The crude estimate of period prevalence will be higher than the actual estimate of point prevalence since it does not allow for individuals with dyskinesia to leave the population during the study period. Closer to our results, Inada and colleagues found a prevalence of TD of 8%, based on psychiatric patients from 11-facilities in Japan [14].

The reason for such a large discrepancy between these rates is not readily apparent. Both the studies used a more precise diagnostic measure for dyskinesia, but only applied it once, thus including individuals who did not meet the Schooler-Kane criteria. In the study by Ganzini and colleagues, 19% of the control group also was reported to have undiagnosed Parkinson’s disease [12]. Selection bias is a likely explanation for the disparity in the results and was not considered by the authors of these studies. Specifically, overestimation of the true association between antipsychotic drug use or MCP and dyskinesia will be greater in studies involving psychiatric patients than for the general population because the association will be evaluated more rigorously in the patient population.

Our findings provide evidence for an independent association between TD and each of the health conditions (diabetes mellitus, psychoses, and especially schizophrenia). This is consistent with previous studies examining the association between these health conditions and TD [15,16], as well as with SD where antipsychotic drugs were not used [8-10]. The greater than 30-fold increased risk of TD among those with schizophrenia (and exposure to an antipsychotic drug) compared with those without schizophrenia is congruent with what many others have reported previously. The estimate is much higher than that reported by Morgenstern and colleagues [4]. We do not have an explanation for this difference. Our findings also provide evidence for an independent association between each of the selected health conditions and SD. The finding of a 4-fold increased risk of spontaneous dyskinesia among persons with schizophrenia (but no exposure to an antipsychotic drug) compared with those without schizophrenia may be a result of the underlying disease, as suggested by others [8-10].

Our study reports data from a defined population, all with health insurance, and coverage for prescription medication. We ascertained the incidence of the SD and TD using this system. There were no changes in the medical coverage of this population during the 10 years of the study that could bias our findings. Our definition of a new case of SD or TD meets minimal standards for such a diagnosis, two episodes separated by up to a year. Because of the nature of the data base individual cases could not be confirmed by an independent examination. The most likely outcome of an independent examination using more precise diagnostic criteria such as the Abornormal Involuntary Movement Scale (AIMS) would be to eliminate some of the individuals who we classified as having SD or TD, and lower the incidence rates. For this reason we consider the incidence rates we report to likely overestimate the true incidence of SD or TD in any general population.

Although the results of this study are important, they should be interpreted with some caution. The study is limited in that the information was obtained purely from an insurance database, and not by clinical evaluations. Examination for and documentation of dyskinesia may have been influenced by the fact that the subject was taking antipsychotic drugs. However, selection bias is likely to be much smaller in our study involving the general population than in a select group of psychiatric patients, especially if they were known to have long-term exposure to antipsychotic drugs or MCP. In addition, the nature and distribution of the movement disorder may not have been accurately documented unless the subject was examined by a neurologist, or ideally, a movement disorders specialist. Finally, the data began in 2001 such that we were unable to determine the duration of medication use. Analyses were based on any prior antipsychotic or MCP medication use, as recorded in the database.

## Conclusion

The incidence of spontaneous and tardive dyskinesia is rare in a large, generally non-smoking population. We were able to confirm estimates by others that diabetes mellitis is associated with increased risk of spontaneous dyskinesia. A higher proportion of those with spontaneous dyskinesia present with spasm of the eyelid muscles (blepharospasm) compared to those with tardive dyskinesia who present with orofacial muscular problems.

## Competing interests

The authors have no financial or non-financial competing interests to declare.

## Authors’ contributions

All authors conceptualized this study. RM carried out the statistical analyses. All authors prepared the paper and have read and approved the final manuscript.

## Pre-publication history

The pre-publication history for this paper can be accessed here:

http://www.biomedcentral.com/1471-244X/13/152/prepub
